# Public Awareness and Perception of Family Medicine in Jeddah, Saudi Arabia

**DOI:** 10.7759/cureus.23320

**Published:** 2022-03-19

**Authors:** Bashair M Alshammrani, Renad O Aljuhani, Khadijah M Basaqr, Eman A Bin Mahfouz, Ebtisam M Alhawsawi, Reem Alqahtani

**Affiliations:** 1 Medicine and Surgery, King Abdulaziz University Faculty of Medicine, Jeddah, SAU; 2 Department of Family Medicine, King Abdulaziz University Hospital, Jeddah, SAU

**Keywords:** awarness, family physician (fp), jeddah, saudi arabia, primary health care centers, family medicine

## Abstract

Background

Family medicine is a specialty that provides care for family members' physical, social, and psychological aspects regardless of age, gender, and health conditions. A family physician can manage a wide range of health conditions and prevent various diseases. However, there are scarce data on the awareness and perceptions of the Saudi population regarding family medicine; therefore, we aim to determine the awareness and perception of family medicine and family physicians in the population of Jeddah, Saudi Arabia.

Methodology

This cross-sectional study was conducted in Jeddah and randomly enrolled 519 participants aged 18 years and older through an online self-administered survey. Microsoft Excel and SPSS were employed for the data entry and analysis.

Result

The analysis indicated that 86.7% of the participants had heard about family physicians, 55.1% recognized the family physician's role, 61.7% had never visited one, and 57.2% were unfamiliar with the locations of family medicine clinics.

Conclusion

The results of this study demonstrated that the general population of Jeddah has moderate positive responses toward the role of a family physician as a vital element in the healthcare system. However, the majority of the participants had never previously visited a family physician and were unfamiliar with the locations of family medicine clinics.

## Introduction

Family medicine is a broad specialty that provides continuing and complete physical, social, and psychological healthcare to individuals and families regardless of gender, age, and medical condition [1]. A family physician is a specialist who can diagnose, treat, prevent, and rehabilitate various acute and chronic diseases in the same patient, providing these services to primary, secondary, and tertiary care centers, although mostly in primary care [2]. One of the family physician’s most critical roles is providing adequate healthcare resources by communicating with other healthcare professionals, assuming an advocacy role for patients when needed [3].

Primary healthcare centers (PHCs) played a vital role in the healthcare system and were introduced worldwide after the Alma-Ata Declaration of 1978, which global healthcare leaders established to fulfill the goal of better health for all [4]. In Saudi Arabia, the first PHC was established in 1980; currently, there are nearly 2,257 such centers [2,5], with approximately 60% located in rural sites, making them cost-effective, equitable, and easy to reach for all communities [1]. FM Residency training programs were first initiated in King Saud University and King Faisal University in 1991 [6], and later in 1995, the Saudi Board was established under the authority of the Saudi Commission for Health Specialties (SCFHS). The program includes three years of systematic instruction covering a wide range of skills and knowledge [7].

Current evidence suggests that one of the significant worldwide challenges is correcting the improper and excessive use of emergency departments by individuals seeking non-urgent care, a finding identified in a study in Jeddah, Saudi Arabia [8]. Patients usually tend to avoid PHCs in favor of secondary and tertiary healthcare centers [9]. This situation indicates that the concept of the family medicine specialty is still ambiguous for community members.

Several international studies have examined the public’s awareness and knowledge of family medicine and family physicians [10,11]. In 2004, a report from Pakistan indicated that many patients who visit specialist physicians are unaware of the role of family physicians in the healthcare system [12]. In Saudi Arabia, a couple of studies conducted in the Jazan and Dammam regions reported a positive perception by the public regarding family medicine and family physicians and these physicians’ influential role in the healthcare system [9,13].

Scarce data address the level of awareness and perceptions of family medicine among the Saudi population. Considering that Jeddah is the second-largest city in Saudi Arabia and to meet the expectations of the Saudi Arabia 2030 national vision for enhancing family medicine and therapeutic and preventive healthcare services [14], we aimed to determine the awareness and perception of family medicine and family physicians in the population of Jeddah.

## Materials and methods

Study design and setting

This was an observational cross-sectional study conducted in the city of Jeddah, Saudi Arabia, from October 4, 2021, to January 18, 2022.

Participant selection

The study included male and female adults, aged 18 years and older, living in Jeddah. We excluded healthcare professionals, individuals younger than 18 years, and individuals from other Saudi regions and provinces. Given that the population size of the city of Jeddah was 4,967,112 at the time of the study, the required sample size was 385 participants for a 95% confidence level and a margin error of 5%. The calculations were performed using the Raosoft sample size calculator (www.raosoft.com/samplesize.html).

The study researchers posted an Arabic version of the questionnaire on numerous social media platforms (Twitter, Telegram, and WhatsApp), and the participants were chosen using a self-selection sampling technique. We employed a reliable self-administered questionnaire, which was taken from another study after obtaining permission from the main author [13]. The researchers then modified the questionnaire, after which a pilot study was conducted to validate the translated questionnaire and ensure its internal consistency using Cronbach’s alpha coefficient (α > 0.98). The Arabic online self-administrated questionnaire consisted of three main sections: (1) demographic data (age, gender, educational status, work, marital status, and monthly income), (2) questions to assess the knowledge of family medicine and the role of family physicians, and (3) a question with a yes/no answer to determine the respondent’s perception of the importance of family physicians.

The purpose of the study was explained to the participants, and their consent was obtained before taking the questionnaire.

Data analysis

We used Microsoft Excel Version 2020 (Microsoft Corporation, Redmond, WA) to enter the data and performed the statistical analysis using IBM SPSS Version 21 (IBM Corp., Armonk, NY). We calculated the mean and standard deviation to describe the continuous variables, and used numbers and percentages for the categorical variables. A p-value of <0.05 was considered statistically significant.

Ethical approval 

Ethical approval was granted by the Biomedical Ethical Committee at King Abdulaziz University Hospital, Jeddah, Saudi Arabia (Reference No. 474-21).

## Results

**Figure 1:**
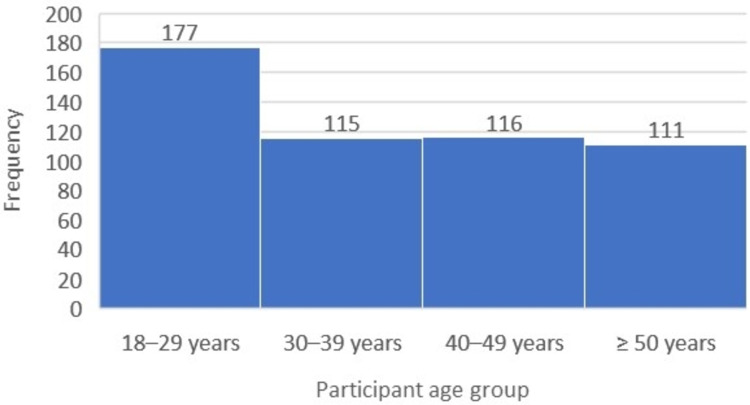
Participant age groups

A total of 519 participants from Jeddah city were enrolled in the study, more than half of whom were female (337, 64.9%). The most frequent age group was 18-29 years (177, 34.1%), while the least frequent age group was >50 years (111, 21.4%), with a calculated mean of 37.08 ± 12.42 (Figure 1).

Most participants were working toward or had obtained a bachelor's degree (308, 59.3%). In terms of monthly income, 201 (38.7%) participants earned less than 1,332 USD, while 94 (18.1%) earned more than 3,998 USD. The demographic characteristics are shown in Table 1.

**Table 1:** Socio-demographic data of the respondents (n=519)

Characteristics of participants		Frequency (%)
Gender		
Female		337 (64.9)
Male		182 (35.1)
Age groups		
18–29 years		177 (34.1)
30–39 years		115 (22.2)
40–49 years		116 (22.4)
50 or more		111 (21.4)
Working status		
Working		209 (40.3)
Not working		310 (59.7)
Marital status		
Married		318 (61.3)
Separated	23 (4.4)	
Single	171 (32.9)	
Widow	7 (1.3)	
Educational status		
Primary school	19 (3.7)	
High school	163 (31.4)	
College	308 (59.3)	
PhD	29 (5.6)	
Family income		
Less than 1,332 USD	201 (38.7)	
1,335–2,665 USD	129 (24.9)	
2,666–3,998 USD	95 (18.3)	
More than 3,998 USD	94 (18.1)	

For the questions regarding family medicine and the role of the family physician, 450 (86.7%) of the studied participants had heard about family physicians, and 286 (55.1%) were aware of the role of the family physician in society; however, more than half (320, 61.7%) had never visited one. When asked if they were familiar with the locations of family medicine clinics, more than half of the sample (297, 57.2%) answered “no” (Table 2).

**Table 2:** Participants’ knowledge and awareness of the specialty of family medicine and the role of family physicians.

	No, N (%)	Yes, N (%)
Have you ever heard about a family physician?	69 (13.3)	450 (86.7)
Have you ever visited a family physician?	320 (61.7)	199 (38.3)
Do you know the role of the family physician in society?	233 (44.9)	286 (55.1)
Do you know the places of family medicine clinics?	297 (57.2)	222 (42.8)

When asked if they were aware of the role of family physicians in treating the chronic diseases hypertension, diabetes, and hyperlipidemia, 386 (74.4%), 369 (71.1%), and 340 (65.5%) respondents answered “yes”, respectively; the rest of the chronic diseases are listed in Table 3. In terms of the family physicians’ role in treating respiratory, skin, and urinary diseases, almost half of the participants answered yes (297, 57.2%; 259, 49.9%; and 235, 45.3%, respectively). In addition, 369 (71.1%) participants considered that the family physician plays a role in the early detection of any disease that can threaten society. Of the participants, 446 (85.9%) recognized that family physicians play a role in helping families follow healthy lifestyles by advising them on exercise and methods for preventing numerous diseases. Most of our study participants agreed that family physicians can help protect families against various diseases by providing them the necessary vaccinations, clinical examinations, and important investigations (369, 71.1%; 361, 69.6%; and 400, 77.1%, respectively).

Our analysis showed that most participants agreed that family physicians have a role in patient follow-up (403, 77.6%) and refer patients to other specialties when needed (437, 84.2%). The number of participants who acknowledged the presence of family physicians in maternity, pediatric health, and preventive medicine was 212 (40.8%), 314 (60.5%), and 325 (62.6%), respectively. More than half of the sample realized that the family physician plays a coordinating role in working with physiotherapists, psychiatrists, and nursing staff (298, 57.4%; 328, 63.2%; and 292, 56.3%, respectively). A majority of the participants (407, 78.4%) were aware that family physicians play a role in maintaining patient records. Furthermore, 437 (84.2%) understood that family physicians maintain their patients’ confidentiality (Table 3).

**Table 3:** Participants’ knowledge and awareness of the specialty of family medicine and the role of family physicians.

	I don’t know, N (%)	No, N (%)	Yes N, (%)
Q1: A family physician has a role in helping family members get environmental care easily.	231 (44.5)	18 (3.5)	270 (52)
Q2: A family physician has a role in treating many of the health problems experienced by family member, such as the following:			
Clinical and surgical disease	(38.7) 201	(22.4) 116	202 (38.9)
Respiratory system diseases	(29.3) 152	(13.5) 70	297 (57.2)
Urinary system diseases	(37.6) 195	(17.1) 89	235 (45.3)
Skin diseases	(34.5) 179	(15.6) 81	259 (49.9)
Q3: A family physician has a role in the early detection of any disease threatening society.	(25.8) 134	(3.1) 16	(71.1) 369
Q4: A family physician has a role in keeping records for each patient, which records his health status and his medical history.	93 (17.9)	19 (3.7)	407 (78.4)
Q5: A family physician has a role in maintaining the confidentiality of his patients and not to disclose to anyone.	76 (14.6)	6 (1.2)	437 (84.2)
Q6: A family physician has a role in advising family members to follow a healthy life, exercise, and learn ways to prevent various diseases.	(13.3) 69	(0.8)4	(85.9)446
Q7: A family physician has a coordinating role in working with the following:			
Physiotherapist	69 (13.3)	(7.9)41	298 (57.4)
Psychiatrists	168 (32.4)	23 (4.4)	328 (63.2)
Nursing staff	186 (35.8)	41 (7.9)	292 (56.3)
Q8: A family physician has a role in protecting family members from various diseases by			
Providing them with the necessary vaccinations	115 (22.2)	35 (6.7)	369 (71.1)
Perform clinical examination	127 (24.5)	31 (6)	361 (69.6)
Ordered important investigations	103 (19.8)	16 (3.1)	400 (77.1)
Early detection of diseases	122 (23.5)	16 (3.1)	381 (73.4)
Q9: A Family physician has a role in the treatment of chronic diseases such as the following:			
Asthma	138 (26.6)	72 (13.9)	309 (59.5)
Diabetes	107 (20.6)	43 (8.3)	369 (71.1)
Hypertension	102 (19.7)	31 (6)	386 (74.4)
Hyperlipidemia	134 (25.8)	45 (8.7)	340 (65.5)
Heart diseases	162 (31.2)	98 (18.9)	259 (49.9)
Thyroid diseases	166 (32)	86 (16.6)	267 (51.4)
Psychiatric diseases	173 (33.3)	79 (15.2)	267 (51.4)
Q10: A family physician is in the following clinics:			
Maternity clinics	197 (38)	110 (21.2)	212 (40.8)
Healthy children clinics	144 (27.7)	61 (11.8)	314 (60.5)
Preventive medicine clinics	164 (31.6)	30 (5.8)	325 (62.6)
Q11: A family physician has a role in the follow-up of the patients’ condition and all the changes that occur.	86 (16.6)	30 (5.8)	403 (77.6)
Q12: A family physician has a role in the referral of the patients to the needed specialist in the case of the inability to treat.	76 (14.6)	6 (1.2)	437 (84.2)

**Figure 2:**
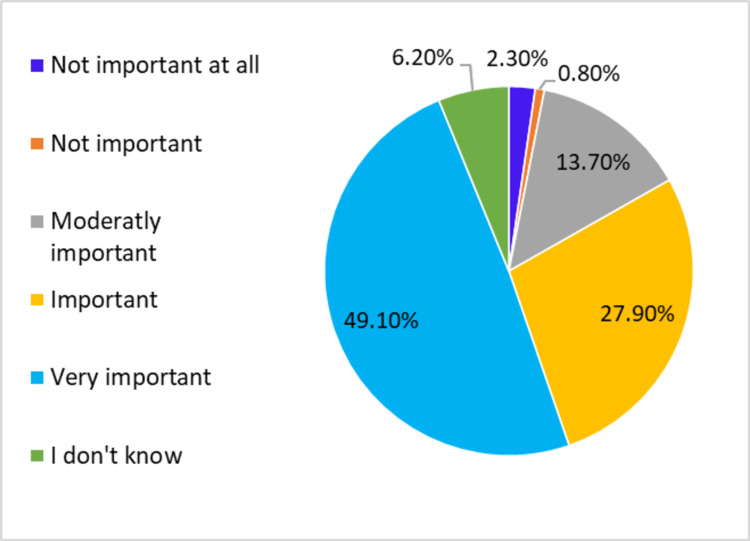
Participants’ assessment of the importance of family physicians for the community. Most of the participants responded “very important” or “important”.

When asked to assess the level of importance of family physicians in society, almost half of the participants (49.10%) stated that family physicians play an essential role in society; however, a minority (2%) believed that family physicians’ role is completely unimportant (Figure 2).

In terms of the association between the participants’ age and family physician visits, the older participants were significantly more likely to visit family physicians than the younger participants (p = 0.020) (Table 4).

**Table 4:** Association between age groups and having visited a family physician, awareness of family physicians’ role, and knowledge of the locations of family medicine clinics.

	Age group				p-Value
	18-29 years	30-39 years	40-49 years	50 years and more	
Have you visited a family physician before?					
Yes	29.4%	40.9%	42.2%	45.9%	0.020
No	70.6%	59.1%	57.8%	54.1%	
Are you aware about the role of family physicians in the community?					
Yes	47.5%	60%	56%	61.3%	0.005
No	52.5%	40%	44%	38.7%	
Are you aware of the places of family medicine clinics?					
Yes	33.3%	41.7%	48.3%	53.2%	0.071
No	66.7%	58.3%	51.7%	46.8%	

Surprisingly, we found that education and monthly income had no effect on the awareness of family physicians’ role in society (p = 0.089 and p = 0.416, respectively) in the current study (Tables 5, 6).

**Table 5:** Association between educational status and having visited a family physician, awareness of family physicians’ role, and knowledge of the locations of family medicine clinics.

	Education				p-Value
	Primary	High	Bachelor's degree	Master's degree or PhD	
Have you visited a family physician before?					
Yes	21.1%	29.4%	41.9%	62.1%	0.001
No	78.9%	70.6%	58.1%	37.9%	
Are you aware about the role of family physicians in the community?					
Yes	63.2%	62.6%	51.3%	48.3%	0.089
No	36.8%	37.4%	48.7%	48.3%	
Are you aware of the places of family medicine clinics?					
Yes	42.1%	38.7%	43.8%	55.2%	0.377
No	57.9%	61.3%	56.2%	44.8%	

**Table 6:** Association between monthly income and having visited a family physician, awareness of family physicians’ role, and knowledge of the locations of family medicine clinics.

	Monthly income				p-Value
	Less than 1,332 USD	1,335–2,665 USD	2,666–3,998 USD	More than 3,998 USD	
Have you visited a family physician before?					
Yes	30.8%	36.4%	47.4%	47.9%	0.008
No	69.2%	63.6%	52.6%	52.1%	
Are you aware about the role of family physicians in the community?					
Yes	54.7%	50.4%	55.8%	61.7%	0.416
No	45.3%	49.6%	44.2%	38.3%	
Are you aware of the places of family medicine clinics?					
Yes	36.3%	40.3%	47.4%	55.3%	0.014
No	63.7%	59.7%	52.6%	44.7%	

According to chi-square analysis, we found a significant association between the participants’ perception toward the importance of family physician in the community and family physician visits; participants who answered very important were more likely to visit family physicians than participants who answered not important (p = 0.000) (Table 7). Moreover, a significant association was found between perception about the importance of family physician and awareness about the role of family physician in the community (p = 0.000) (Table 7). In terms of the association between the participants’ perception toward the importance of family physician in the community and knowing the places of family medicine clinics, participants who answered very important were significantly more likely to know the places of family medicine clinics compared to participants who answered not important (p = 0.000) (Table 7).

**Table 7:** Association between assessment of the importance of family physicians for the community and having visited a family physician, awareness of family physicians’ role, and knowledge of the locations of family medicine clinics.

	Level of importance of family physicians for the community						p-Value
	Not important at all	Not important	Moderately important	Important	Very important	I don’t know	
Q1: Have you visited a family physician before?							
Yes	66.7%	25%	21.1%	30.3%	48.2%	25%	0.000
No	33.3%	75%	78.9%	69.7%	51.8%	75%	
Q2: Are you Aware about the role of family physicians in the community?							
Yes	41.7%	25%	45.1%	48.3%	68.2%	12.5%	0.000
No	58.3%	75%	54.9%	51.7%	31.8%	87.5%	
Q3: Are you Aware of the places of family medicine clinics?							
Yes	41.7%	50%	23.9%	34.5%	57.6%	3.1%	0.000
No	58.3%	50%	76.1%	65.5%	42.4%	96.9%	

## Discussion

The importance of family medicine has been growing in Saudi Arabia, as it requires family physicians due to the increased incidence of chronic disease [15]. Family medicine is a specialty that provides continuing care for family members regardless of age, gender, or comorbidities. Family medicine treats all patients with acute illness and chronic diseases such as diabetes, hypertension, and asthma. Therefore, we aimed to investigate the public’s perception and awareness of family medicine and family physicians. The analysis showed that nearly half of the participants (286, 55.1%) were aware of the role of family physicians, and most respondents (450, 86.7%) had previously heard about family physicians. However, a large percentage of the participants (61.7%) had never visited a family physician, and fewer than half knew the locations of family medical centers. These outcomes are similar to another study conducted in Jazan, which reported that 467 (56.3%) of the participants were aware of the role of family physicians. However, a large group of the participants (749, 90.2%) had no regular family physician, and 496 (59.8%) preferred seeing a specialist [9]. According to a previous study by Aldhamadi and Alzahrani in the cities of Dammam and Al-Khobar, the reasons for avoiding PHCs might include long waiting times (29.94%), inconvenient working hours (29.94%), unreliable physicians (29,38%), and distant PHCs (6.21%) [16]. In another study in Dammam, Bograin et al. reported that 61.1% of the study participants know about the role of family physicians in treating clinical and surgical diseases such as respiratory, urinary, and skin diseases [13], results that agree with ours to some extent; almost half of our participants were aware that family physicians could treat respiratory (57.2%), urinary (45.3%), and skin (49.9%) diseases.

One of the family physician’s essential role is protecting family members against various diseases, which can be achieved through vaccinations. Fortunately, a large number of the participants (369, 71.1%) were aware of this fact, a finding that is in keeping with a published article from Nairobi, in which a significant proportion of participants were aware that adult (67%) and pediatric (64%) immunizations are provided by family medicine clinics [17]. Regarding the involvement of family physicians in various clinics such as pediatric health and preventive medicine, the analysis estimated that 40.8% of our study participants knew about the presence of family physicians in maternity clinics, which is lower than the percentage estimated in the previous study from Nairobi, where 55% confirmed that family physicians had a part in prenatal clinics [17].

Another article indicated that most patients skip PHCs, preferring to visit specialized clinics for prenatal and postpartum follow-up [16]. In terms of factors related to family physician visits, we found a significant association with age groups (p = 0.020), in which the older participants were more likely to visit family physicians than the younger participants. This result corresponds with that of the other study conducted in Jazan [9], where older participants were more aware of the role of family physicians in the community than younger participants (p < 0.000), as the incidence of chronic conditions increases with age. In the current study, surprisingly, education and monthly income had no statistically significant effect on the awareness of the role of family physicians in society (p = 0.089 and p = 0.416, respectively).

Most of the participants reported a positive attitude toward the role of family physicians when asked to rate the importance of the physicians’ role in the community: 49.10% answered “very important”, 27.9% answered “important”, and only 2.3% answered “not important at all”. The results are comparable to another research study in Dammam, where 55.7% of the sample agree with the importance of family physicians in the community [13].

Limitation and recommendations

There are a few potential limitations to consider in this study. First, the sampling technique was narrowed to a single city. Therefore, the results cannot be applied to other parts of Saudi Arabia. Secondly, the data were collected through online self-reported questionnaires due to COVID-19 precautions. The earlier findings proved that despite the high awareness regarding family medicine and family physicians, there is a tendency to favor emergency departments and specialized clinics over family medicine clinics. We, therefore, recommend organizing community-based campaigns to emphasize the benefits of a regular physician as the first contact and clarifying the services that family medicine clinics offer. These efforts will ensure complete, easy, and time-saving access to healthcare and will markedly decrease the burden on other medical departments.

## Conclusions

The results of this study demonstrated that the general population of Jeddah has moderate positive responses toward the role of family physician as a vital element in the healthcare system. However, the majority of the participants had never previously visited a family physician and were unfamiliar with the locations of family medicine clinics. It is hoped that educating the public and emphasizing on the role of family physician will increase the awareness and enhance the therapeutic and preventive healthcare services.
